# Supplementary material for: An Inhibitory Role for Human CD96 Endodomain in T Cell Anti-Tumor Responses
Source: Cells. 2023 Jan 13;12(2):309. doi: 10.3390/cells12020309 (PMC9856660; doi:10.3390/cells12020309)
Supplement: Supplementary file 1 [file cells-12-00309-s001.zip › cells-2104242-supplementary.pdf]

Supplementary Material

1 Supplementary Figures

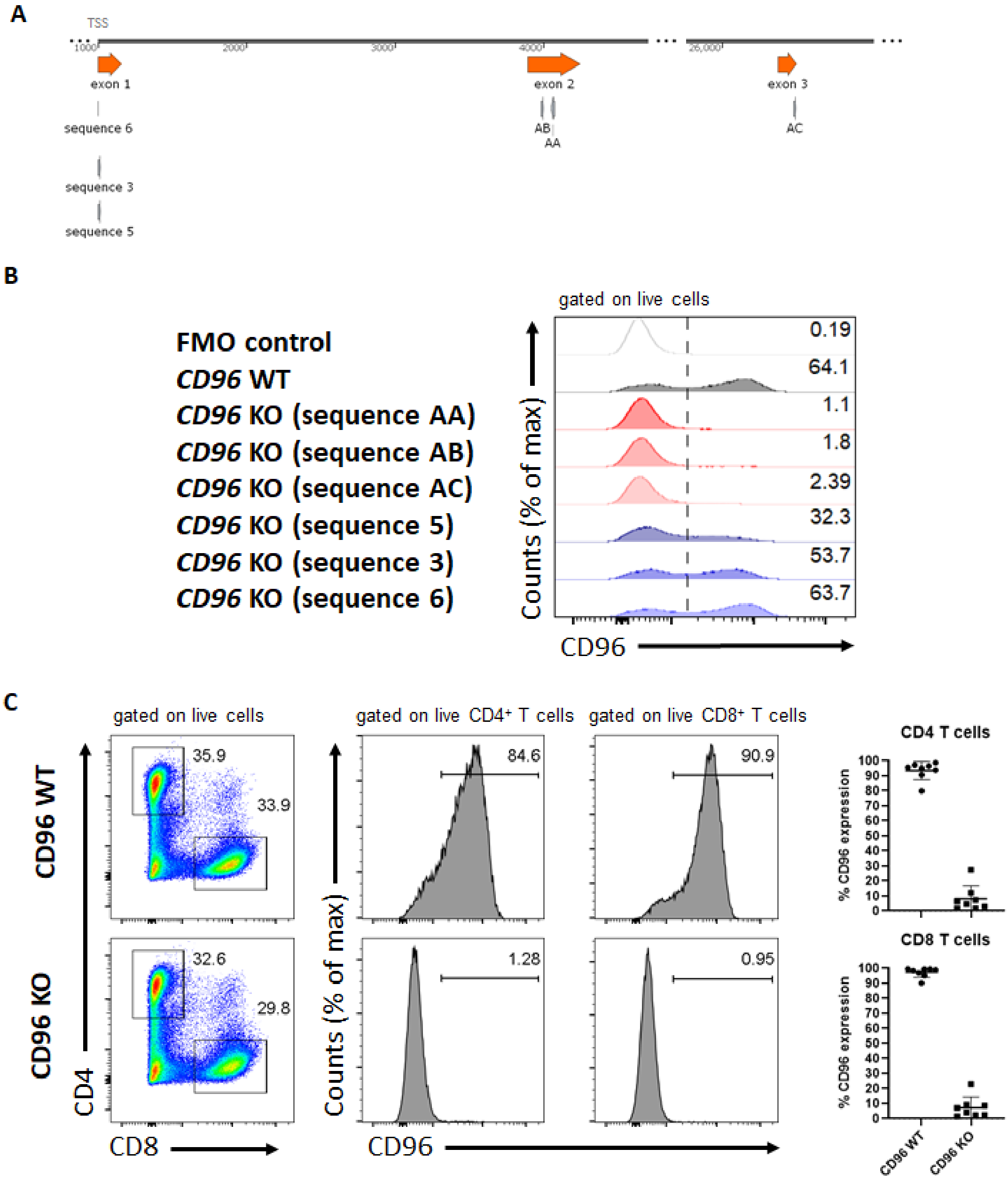

Figure S1. CRISPR/Cas9-mediated deletion of *CD96* in human T cells. (A) Schematic diagram showing part of the *CD96* genomic locus depicting exons which are targeted by the six gRNAs described in (B) for CRISPR/Cas9-mediated *CD96* gene disruption. TSS, transcription start site. Numbers indicate the positions of bases along the *CD96* locus. (B) Expression of CD96 in total T cells activated as in figure 1A, electroporated with various gRNAs (AA, AB, AC and sequences 3, 5 and 6 gRNAs) and Cas9 to generate *CD96* KO T cells or without gRNA to generate *CD96* WT T cells, and expanded for further 3 days. (C) Representative flow cytometry plots showing expression of CD96 in $CD4^+$ and $CD8^+$ T cells electroporated as in (B) with AA gRNA (left). Graphs showing knockout efficiency of CD96 in T cells derived from 5 different donors across 4 independent experiments (right). Data in (B) are representative of 2 independent experiments.

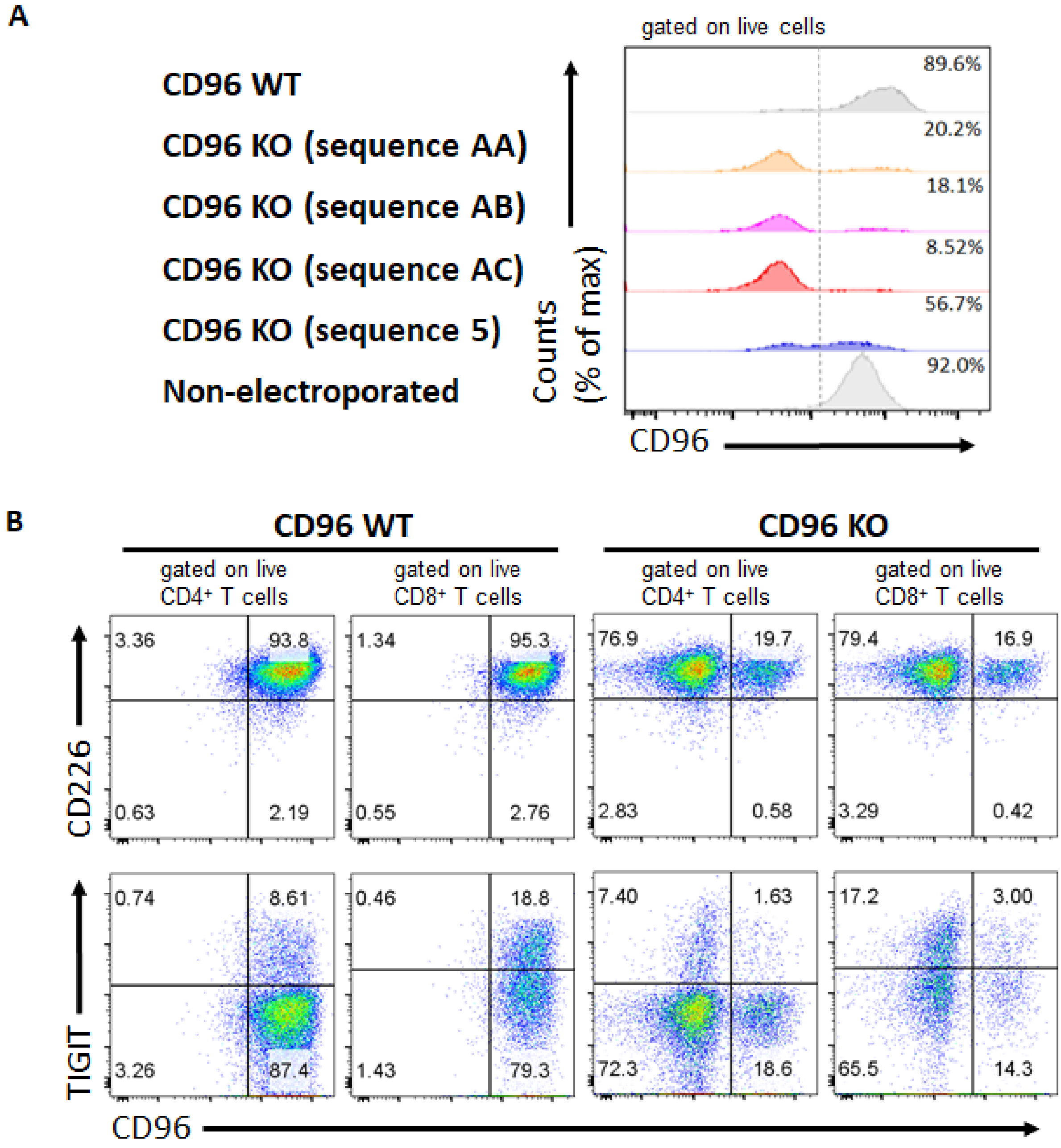

Figure S2. *CD96* deletion did not alter expression of CD96 family receptors CD226 and TIGIT in human T cells. (A) Expression of CD96 in non-electroporated T cells, T cells electroporated with no gRNA (mock-electroporated), or with AA, AB, AC and sequence 5 gRNAs as in figure S1B. (B) Expression of CD226 (top panel) and TIGIT (bottom panel) in viable *CD96* WT and KO $CD4^+$ and $CD8^+$ T cells. Data in (A) are representative of 4 independent experiments. Data in (B) are representative of T cells from at least 4 independent donors assessed in at least 3 independent experiments.

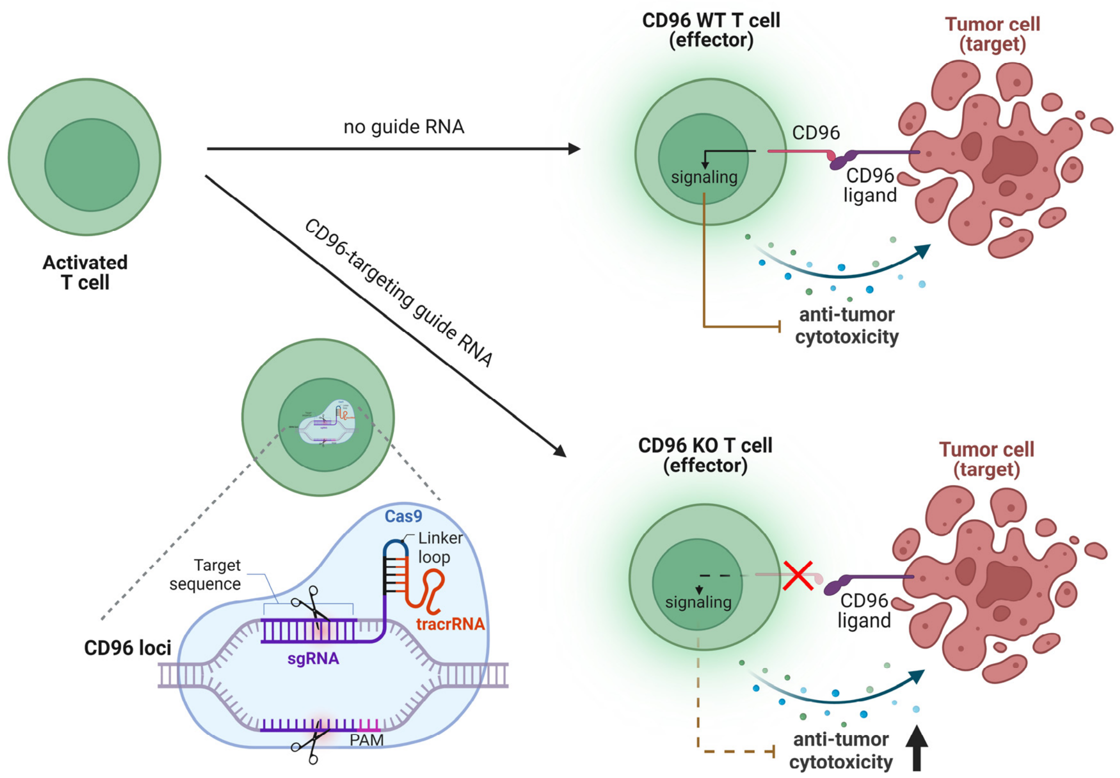

Figure S3. CD96 suppresses the cytotoxicity of T cells against a subset of CD155-expressing tumor cell types. *CD96* was deleted in activated T cells to generate *CD96* KO T cells using CRISPR/Cas9 guide RNAs (gRNAs) to target the *CD96* locus as in figure S1A. Engagement of CD96 on WT T cells with its ligands such as CD155 on tumor cells elicits CD96 signaling that inhibits T cell anti-tumor response (top panel). However, the absence of CD96 in KO T cells relieves such inhibitory signalling and results in enhanced anti-tumor response (bottom panel). This figure is adapted from "The CRISPR-Cas9 Genetic Scissors", by BioRender.com (2023). Retrieved from https://app.biorender.com/biorender-templates.

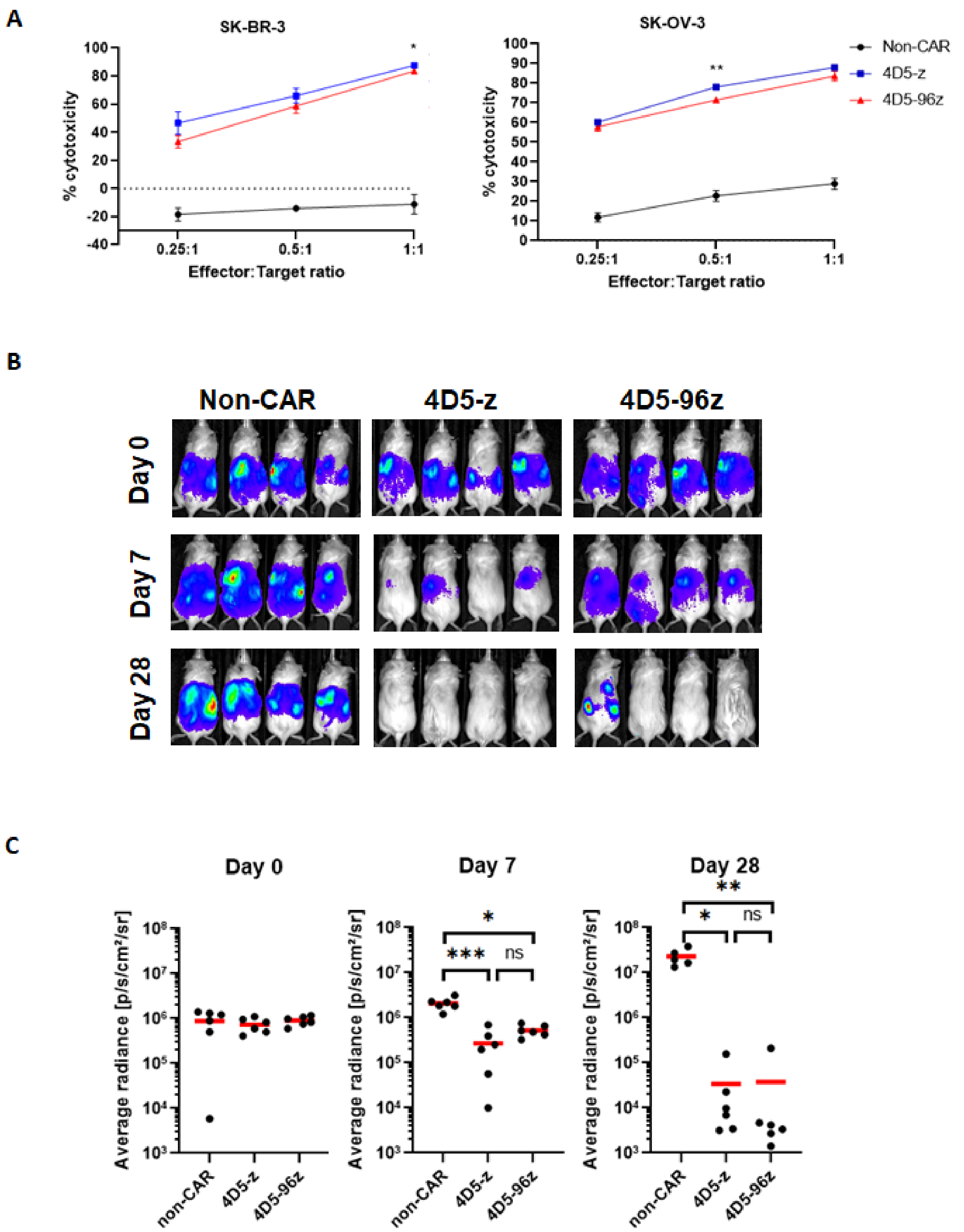

Figure S4. Inclusion of CD96 endodomain in 4D5-z CAR-T cells cripples their cytotoxicity against HER2$^+$ tumor cells *in vitro* and *in vivo*. (A) Percentage (%) cytotoxicity of CAR-T cells against luciferase-expressing SK-BR-3 and SK-OV-3 cells 20 h after co-culture with T cells at indicated E:T ratios (left panels). For clarity, only statistical significance resulting from comparisons of % cytotoxicity of 4D5-z and 4D5-96z CAR-T cells are shown. (B) Selected images of tumor bioluminescence (BLI) at indicated time points (days) prior to and post T cell infusion in the tumor model using SK-OV-3 cells in NSG mice. (C) Average radiance of tumor burden in mice at indicated time points post infusion of non-CAR-T, 4D5-z or 4D5-96z CAR-T cells. Data in (C) are based on 6 mice analyzed with each symbol representing one mouse and red horizontal bars indicating the mean; Kruskal-Wallis test, *, $p < 0.05$; **, $p < 0.005$; ***, $p < 0.001$.

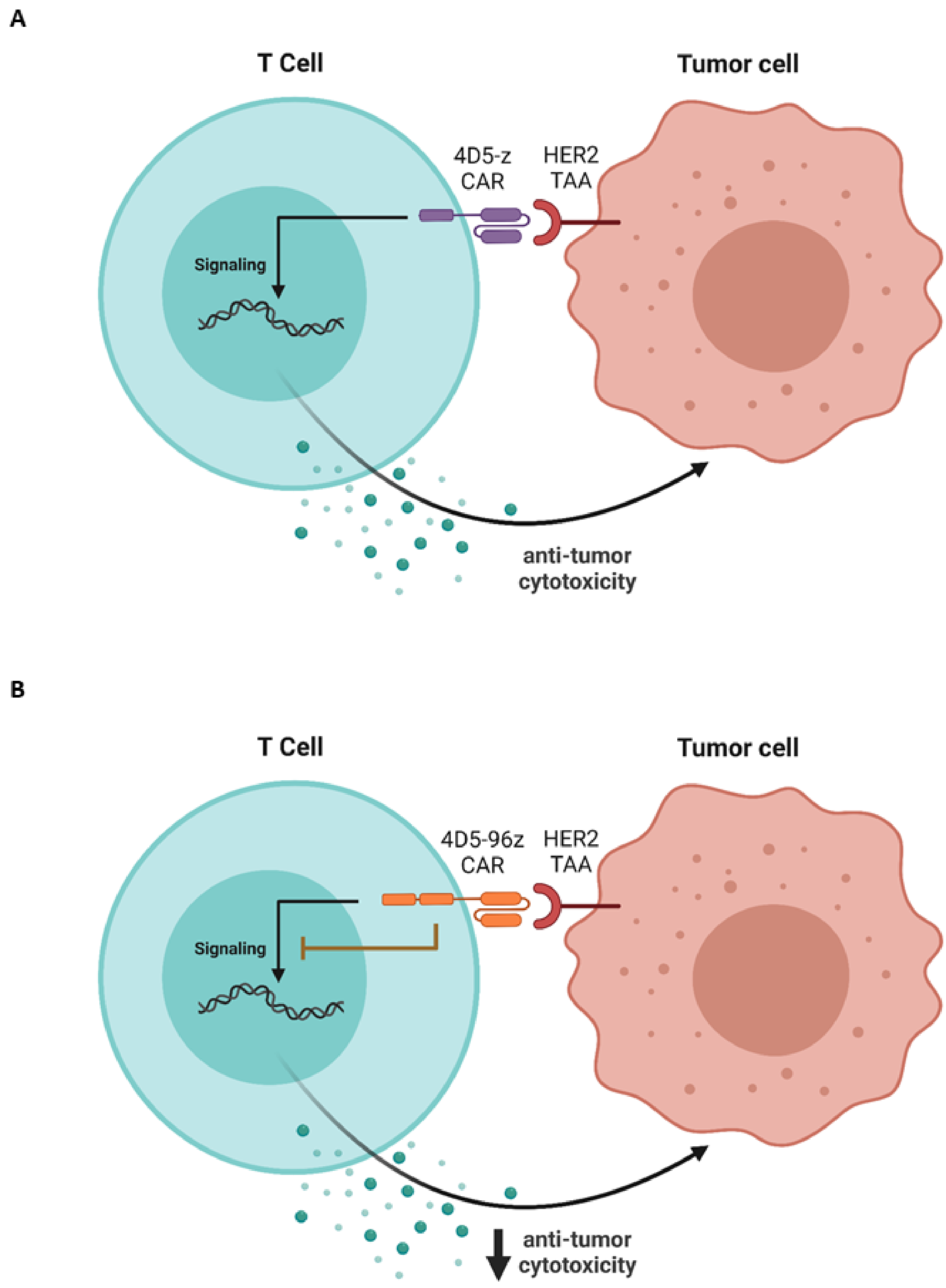

Figure S5. CD96 intracellular signalling (IC) domain plays an inhibitory role in T cell anti-tumor responses. 4D5-z or 4D5-96z CAR-T cells were generated by transducing activated T cells with CAR constructs as in figure 3A. 4D5-z CAR elicits an anti-tumor response in T cells upon engagement with the cognate HER2 TAA on tumor cells. However, incorporation of the CD96 IC domain, which emanates inhibitory signaling by yet unknown mechanism, into 4D5-96z CAR endows it with inhibitory function to attenuate T cell anti-tumor response. Abbreviations: CAR, chimeric antigen receptor; TAA, tumor-associated antigen; IC, intracellular signalling domain. This figure is adapted from "CAR-Engrafted T cell and Tumor Cell", by BioRender.com (2021). Retrieved from https://app.biorender.com/biorender-templates.
